# MaAreB, a GATA Transcription Factor, Is Involved in Nitrogen Source Utilization, Stress Tolerances and Virulence in *Metarhizium acridum*

**DOI:** 10.3390/jof7070512

**Published:** 2021-06-27

**Authors:** Chaochuang Li, Qipei Zhang, Yuxian Xia, Kai Jin

**Affiliations:** 1 Genetic Engineering Research Center, School of Life Sciences, Chongqing University, Chongqing 401331, China; leezc90@163.com (C.L.); zhangqipei12201@yeah.net (Q.Z.); 2 Chongqing Engineering Research Center for Fungal Insecticide, Chongqing 401331, China; 3 Key Laboratory of Gene Function and Regulation Technologies Under Chongqing Municipal Education Commission, Chongqing 401331, China

**Keywords:** *Metarhizium acridum*, GATA transcription factor, MaAreB, nitrogen utilization, stress response, virulence

## Abstract

The nitrogen catabolite repression (NCR) pathway is involved in nitrogen utilization, in which the global GATA transcription factor AreA plays an indispensable role and has been reported in many fungi. However, relatively few studies are focused on AreB, another GATA transcription factor in the NCR pathway and the functions of AreB are largely unknown in entomopathogenic fungi. Here, we characterized MaAreB in the model entomopathogenic fungus *Metarhizium acridum*. Sequence arrangement found that MaAreB had a conserved GATA zinc finger DNA binding domain and a leucine zipper domain. Disruption of *MaAreB* affected the nitrogen utilization and led to decelerated conidial germination and hyphal growth, decreased conidial yield, and lower tolerances to UV-B irradiation and heat-shock. Furthermore, the *MaAreB* mutant (Δ*MaAreB*) exhibited increased sensitivity to CFW (Calcofluor white), decreased cell wall contents (chitin and β-1,3-glucan) and reduced expression levels of some genes related to cell wall integrity, indicating that disruption of *MaAreB* affected the cell wall integrity. Bioassays showed that the virulence of the Δ*MaAreB* strain was decreased in topical inoculation but not in intra-hemocoel injection. Consistently, deletion of *MaAreB* severely impaired the appressorium formation and reduced the turgor pressure of appressorium. These results revealed that *MaAreB* regulated fungal nitrogen utilization, cell wall integrity and biological control potential, which would contribute to the functional characterization of AreB homologous proteins in other insect fungal pathogens, and even filamentous fungi.

## 1. Introduction

There are many fungi in the natural environment; some are pathogenic and are a threat to human health, agriculture and forestry, such as *Aspergillus fumigatus*, *Candida albicans*, *A. flavus*, *Fusarium graminearum*, and *Magnaporthe oryzae* [1,2]. Some are important biological control fungi, which can be used to control crop diseases and pests, such as *Trichoderma harzianum*, *Beauveria bassiana* and *Metarhizium anisopliae* [3,4,5]. Entomopathogenic fungi, as an important class of insecticidal microbes, are important regulators of the insect population in nature, and have been applied to the control of agricultural and forestry pests [6,7]. Similar to many plant fungal pathogens, the conidia of insect fungal pathogens, like *B*. *bassiana*, could adhere to the host cuticles and penetrate these via the infection structure appressorium under the combination of turgor pressure and cuticle-degrading enzymes [8,9,10]. Under natural conditions, fungi have to cope with a variety of environmental challenges, such as temperature, ultraviolet rays, nutritional restriction conditions, etc. [11].

Nitrogen is a necessary influencing factor for fungal survival. Fungi can uptake some kinds of compound as nitrogen sources, but other nitrogen sources will not be assimilated in the presence of preferentially used ammonium or glutamine; if not, they can assimilate other nitrogen sources, such as nitrate and urea [12,13]. This phenomenon of nitrogen utilization is well known as nitrogen metabolite repression (NMR), also called nitrogen catabolite repression (NCR) in *Saccharomyces cerevisiae*, which mainly regulates genes related to nitrogen utilization at the transcription level [12]. In *ascomycetes*, NCR is mediated by GATA transcription factors, and four GATA transcription factors are characterized in *S. cerevisiae*, namely Gln3 and Gat1, which play positive regulatory roles, and Dal80 and Gzf3, which play negative regulatory roles [11]. However, only two GATA transcription factors, AreA and AreB, in the NCR pathway were found in filamentous fungi [14]. The AreA homologous proteins have been characterized in many fungi, such as AreA in *A. nidulans* [15], Nit2 in *N. crassa* [16] and Nut1 in *M. oryzae* [17]. AreA can activate the expression of genes that make full use of non-preferential nitrogen sources to relieve nitrogen repression in the absence of preferential nitrogen sources [18]. Accumulated research has indicated that AreA plays important roles in nitrogen utilization and the growth and development of fungi [19,20,21,22].

AreB is another GATA transcription factor, involved in regulating nitrogen metabolism in *A. nidulans* [23]. AreB is homologous to Dal80 and Gzf3, and may play a negative regulatory role by competing with the promoter binding sites with AreA [14,23]. The overexpression of *areB* can repress the transcription of genes regulated by AreA in *A. nidulans* [14], and the overexpression of *nreB*, a homologous gene of *AreB*, would down-regulate the expression of nitrate assimilation related genes in *P. chrysogenum* [24], indicating that AreB acts as a repressor and negatively regulates the activity of AreA. Furthermore, *mepC* [25] and *nmr1* [26], the AreA-dependent genes, are highly expressed in Δ*AreB* mutant, indicating that AreA and AreB share the same target genes, but with different regulation pathways [18]. Interestingly, *asd4*, a homologous gene of *AreB*, does not regulate nitrogen metabolism, but regulates the development of ascospores in *N. crassa* [27]. In *F. fujikuroi*, AreB can simultaneously play positive and negative regulatory roles in nitrogen metabolism, and both AreA and AreB play important roles in the biosynthesis of gibberellin [11]. In *M. oryzae*, *asd4* is required for nitrogen metabolism including glutaminolysis and loss of *asd4* would result in failure of appressorium formation and abolishment of virulence [28]. To date, however, little is known about the involvement of the AreB homologous protein in potential biological control of entomopathogenic fungi.

In this study, MaAreB, a GATA transcription factor AreB homologous protein, was identified in *M. acridum*. To reveal the underlying roles of *MaAreB*, we characterized *MaAreB* by gene knockout and complementation technology in *M. acridum*. This showed that *MaAreB* not only played important roles in regulating nitrogen utilization, but also in the growth characteristic, stress tolerance, cell wall integrity and virulence of *M. acridum*. These results suggest a multifunctional role of *MaAreB* in development and biological control potential for *M. acridum*.

## 2. Materials and Methods

### 2.1. Strains and Culture Conditions

The *M. acridum* wild-type strain CQMa102 (WT), *MaAreB*-disruption strain (Δ*MaAreB*) and complemented strain (CP) were cultured on 1/4SDAY (0.5% yeast extract, 1% glucose, 0.25% peptone and 1.8% agar, *w*/*v*), Czapek-dox (CZA) (0.001% FeSO_4_, 0.1% K_2_HPO_4_, 0.05% KCl, 0.2% NaNO_3_, 0.05% MgSO_4_, 3% sucrose and 1.8% agar, *w*/*v*), and/or modified CZA medium at 28 °C for hours or days. *E. coli* BGT1 (Solarbio, Beijing, China) was used to construct the recombinant plasmids, and *Agrobacterium tumefaciens* AGL1 (Solarbio, Beijing, China) was used for the genetic transformation of *M. acridum*.

### 2.2. Bioinformatics Analysis

The protein sequences of MaAreB (NCBI reference sequence, MAC_07065) and its homologous proteins were retrieved and downloaded from GenBank (https://www.ncbi.nlm.nih.gov/protein/) (accessed on 3 March 2021). Domains in MaAreB protein were predicted online via SMART interface (http://smart.emblheidelberg.de/) (accessed on 3 March 2021). Multiple sequence alignments were performed by the DNAMAN program, and the neighbor-joining tree was constructed by MEGA6 under the condition of default settings and 1000 bootstrap replicates [29].

### 2.3. Construction of the Mutants

To construct the *MaAreB*-disruption vectors, about 1000-bp up- and down-stream fragments of *MaAreB* were cloned from WT genomic DNA and inserted into pK2-SM-F and pK2-SM-R vectors [30] to form the gene knockout vectors, pK2-SM-*MaAreB*-F and pK2-SM-*MaAreB*-R (Figure S1A). The DNA and promoter sequence of *MaAreB* was amplified and inserted into pK2-sur vector [31] to form the complementation plasmid, pK2-sur-*MaAreB* (Figure S1B). Both knockout and complementation recombinant vectors were transferred into AGL1 for fungal transformation [32]. The Δ*MaAreB* and CP transformants were screened on CZA plates supplemented with 0.5 g/L glufosinate-ammonium (Sigma, St. Louis, MO, USA) or 0.02 g/L chlorimuron ethyl (Sigma, Bellefonte, PA, USA), respectively. Firstly, the fragments of target gene and resistance genes were amplified for preliminary verification of the transformants. On the other hand, Southern blotting was used for further verification via DIG High Prime DNA Labeling and Detection Starter Kit I (Roche, Basel, Switzerland) (Figure S1C). All primers are listed in Table S1.

### 2.4. Growth Characteristic Assays

The conidia of WT, Δ*MaAreB* and CP strains, cultured on 1/4SDAY for 15 days, were used for preparation of conidial suspensions (10^6^ or 10^7^ conidia/mL). For examination of the conidial germination rate and observation of hyphal morphological development, 100 μL conidial suspensions (10^7^ conidia/mL) were pipetted and evenly coated on 1/4SDAY plate, followed by incubating for hours, then the germination of conidia was counted every two hours [33], and photographs taken every three hours, to record the mycelial growth, with a digital light microscope (MOTIC, Xiamen, China). For determination of the conidial yield, 2 μL conidial suspensions (10^6^ conidia/mL) were spotted onto 1/4SDAY or modified CZA medium and cultured at 28 °C for days, and every colony was broken in 1 mL 0.1% Tween-80 to count the number of conidia with a hemocytometer on different days [33]. To analyze the nitrogen utilization, we improved the CZA medium and replaced nitrate with 10 mM (NH_4_)_2_SO_4_, Glutamine, Glutamate or Proline to act as the sole nitrogen source, respectively. Two microliters of conidial suspensions (10^6^ conidia/mL) were spotted onto modified CZA medium and cultured at 28 °C for 7 days to observe the colony morphology and determine the conidial yield.

### 2.5. Stress Tolerance Assays

The tolerances of *M. acridum* to UV-B and heat-shock resistance were tested as described previously [34]. Briefly, in the UV-B tolerance test, 50 μL conidial suspensions (10^7^ conidia/mL) of different fungal strains were evenly spread on 1/4SDAY plates, followed by exposure to UV-B irradiation (1350 MW/m^2^) for 1.25 h, 2.50 h, 3.75 h and 5.00 h, which provided total doses of 6.075, 12.150, 18.225, 24.300 kJ/m^2^, respectively. A 40-W fluorescent lamp was used to provide the UV-B irradiation, and the solid plates were evenly distributed side by side on both sides under the lamp. For heat-shock tolerance test, the conidial suspensions (10^7^ conidia/mL) of each strain were treatment in a 45 °C water bath for hours, followed by spreading 50 μL evenly on 1/4SDAY every three hours. All strains treated with UV-B irradiation and heat-shock were cultured for 20 h to examine the conidial germination.

To analyze cell wall integrity, 2 μL conidial suspensions (10^6^ conidia/mL) of each strain were spotted on 1/4SDAY (control) or supplemented with 150 μg/mL cell wall perturbing agent CFW, then incubated for 7 days at 28 °C, followed by observation of the colony growth and calculation of the relative inhibition rates [35].

### 2.6. Determination of Cell Wall Composition

The cell wall contents, chitin, β-1,3-glucan and mannoproteins, of *M. acridum* were determined according to the previous study [36]. Briefly, hyphae of the WT, Δ*MaAreB* and CP strains were respectively inoculated in a 40 mL 1/4SDY liquid medium (1/4 SDAY without agar) and cultured at 28 °C with 220 rpm for 3 days. Then, the hyphae were harvested and washed with 2% SDS four times, followed by lyophilizing for 24 h. Five milligrams (dry weight) hyphae were consumed for each determination. For determination of the chitin content, hydrochloric acid was used to hydrolyze fungal cell wall, and glucosamine was used as the standard. For determination of β-1,3-glucan content, sodium hydroxide was used to treat the fungal cell wall to obtain the alkali-insoluble fraction, followed by degrading with zymolyase 100-T, and laminaripentaose (Sigma, St. Louis, MO, USA) was used as the standard. To detect the content of mannoprotein, sodium hydroxide was used to extract the mannoprotein in each strain. The standard curve of bovine serum albumin was made via Folin-phenol reagent method.

### 2.7. Quantitative Reverse Transcription PCR (qRT-PCR) Analysis

The mature (15-day-old) conidia on 1/4SDAY medium were harvested and used to detect the expression of genes involved in the tolerances to UV-B, heat-shock, or the cell wall integrity. Appressoria of each strain were collected from the locust hind wings after 27 h of incubation at 28 °C to detect the expression of genes involved in fungal adhesion, cuticle penetration, or glycerol synthesis. The RNAs of fungal cells (conidia and appressoria) were extracted with fungal RNA Kit (CoWin Biosciences, Beijing, China). PrimeScript^TM^ RT reagent Kit with gDNA Eraser (TaKaRa, Dalian, China) was used for synthesising cDNA. qRT-PCR was performed using SYBR^®^ Premix Ex TaqTM (TaKaRa, Dalian, China). *M. acridum gpdh* (EFY84384) acted as the reference gene, and the data were analyzed according to the 2^−ΔΔCt^ method [37]. Forty cycles were performed for all experiments. The details of CT value, amplicon length, and PCR efficiencies for each primer pair are provided in supplementary Table S2. The primers used for qRT-PCR are listed in supplementary Table S1.

### 2.8. Insect Bioassays

Topical inoculation and intra-hemocoel injection methods were used to investigate the virulence of the WT, Δ*MaAreB* and CP strains as described previously [38]. Briefly, 5 μL 10^7^ conidia/mL conidial suspensions were dropped on the pro-nota of a fifth-instar locust nymph as topical inoculation test, and liquid paraffin acted as control. Five microliters 10^6^ conidia/mL of conidial suspensions were injected into the hemolymph of a fifth-instar locust nymph as intra-hemocoel injection test, and sterile water was used as the control. The bioassay environment was maintained in a stable photoperiod (16-h light and 8-h dark) with an ambient temperature of 28 °C. Three replicates of each treatment (*n* = 30 locusts) were performed, and the experiment was repeated three times. The mortality was recorded every half day until all the test insects had died. Virulence of each strain was estimated with the LT_50_ (the mean 50% lethality time). In order to observe the growth of *M. acridum* in locust hemolymph, 600 μL blood for each treatment were collected to count the number of hyphal bodies and quantify the gDNA concentrations of the *M. acridum*, using quantitative PCR with the specificity ITS primers [39].

### 2.9. Infection Structure Development Assays

Conidial germinations and appressorium formations of the WT, Δ*MaAreB* and CP strains were determined as previously described [40]. Appressoria from different fungal strains were treated with different concentrations of PEG8000 to analyze the appressorial turgor pressure. The neutral lipids were stained with Nile Red as described previously [41], and the fluorescence intensity of the neutral lipids in appressorium was measured by ImageJ software.

### 2.10. Statistical Analysis

The data were presented as the mean ± SD and analyzed using ANOVA (one-way analysis of variance) with the SPSS 24.0 program. The differences were analyzed with Tukey’s HSD test. Three biological replicates were performed for each result.

## 3. Results

### 3.1. Bioinformatics Analysis and Generation of MaAreB Mutants

**Figure 1:**
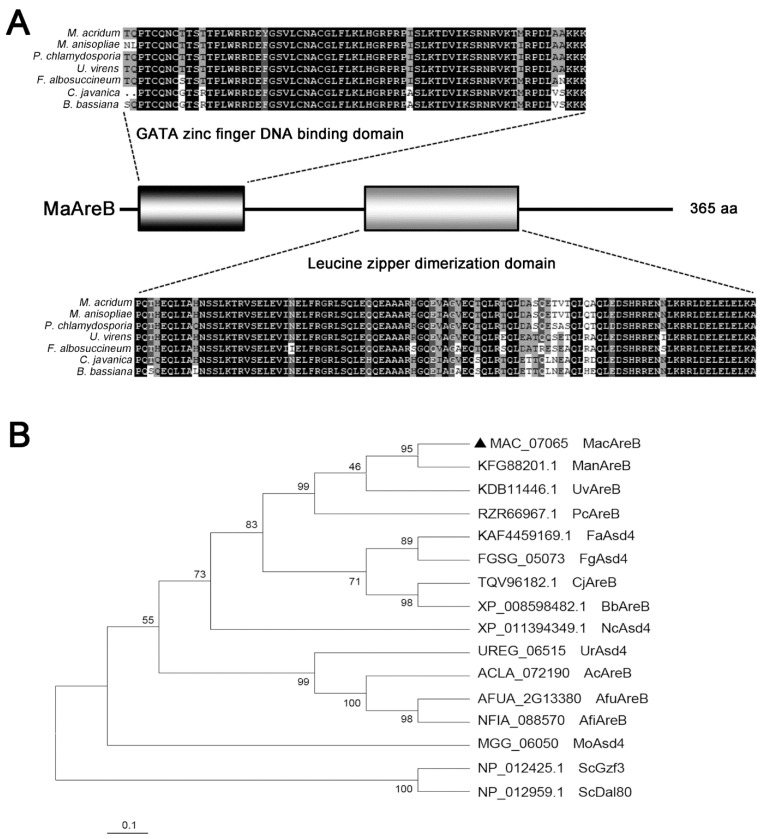
Sequence and phylogenetic analysis of MaAreB. (**A**) Conserved domain arrangements of the MaAreB and its orthologues in other fungal species. (**B**) Phylogenetic tree analysis of AreB proteins in multi-species. AreB, Asd4, Gzf3 and Dal80 are orthologues. *Mac*, *Metarhizium acridum*. *Man*, *Metarhizium anisopliae*. *Uv*, *Ustilaginoidea virens*. *Pc*, *Pochonia chlamydosporia*. *Fa*, *Fusarium al-bosuccineum*. *Fg*, *Fusarium graminearum*. *Cj*, *Cordyceps javanica*. *Bb*, *Beauveria bassiana*. *Nc*, *Neurospora crassa*. *Ur*, *Uncinocarpus reesii*. *Ac*, *Aspergillus clavatus*. *Afu*, *Aspergillus fumigatus*. *Afi*, *Aspergillus fischeri*. *Mo*, *Magnaporthe oryzae. Sc*, *Saccharomyces cerevisiae*. The triangle represented the AreB homologous protein in *M. acridum*.

*MaAreB* was cloned from the genomic DNA of the WT strain; the DNA sequence was 1152 bp with one intron. The ORF (open reading frame) was 1098 bp, encoding 365 amino acids. The physical and chemical properties of MaAreB were analyzed in silico, showing that the protein mass was 39.77 kDa, and the isoelectric point was 4.76. *MaAreB* encoded a GATA transcription factor with a zinc finger DNA binding domain at the N-terminal and a leucine zipper domain at the C-terminal. Both domains were highly conserved in fungi, and the identity was 51.30% by protein sequence alignment of MaAreB with its homologs (Figure 1A). The homologous proteins of AreB, such as Asd4, Gzf3, or Dal80, were found in many fungi, and the phylogenetic tree analysis showed that MaAreB was relatively close to the homologous proteins of filamentous fungi in evolution, and far from yeast (Figure 1B). 

According to the principle of homologous recombination, the Δ*MaAreB* strain was obtained by replacing the 1.1 kb *MaAreB* gene with the *bar* cassette (Figure S1A). The CP strain was constructed by the principle of random insertion (Figure S1B). All the fungal strains were verified by Southern blotting (Figure S1C).

### 3.2. MaAreB Was Involved in the Regulation of Nitrogen Utilization

**Figure 2:**
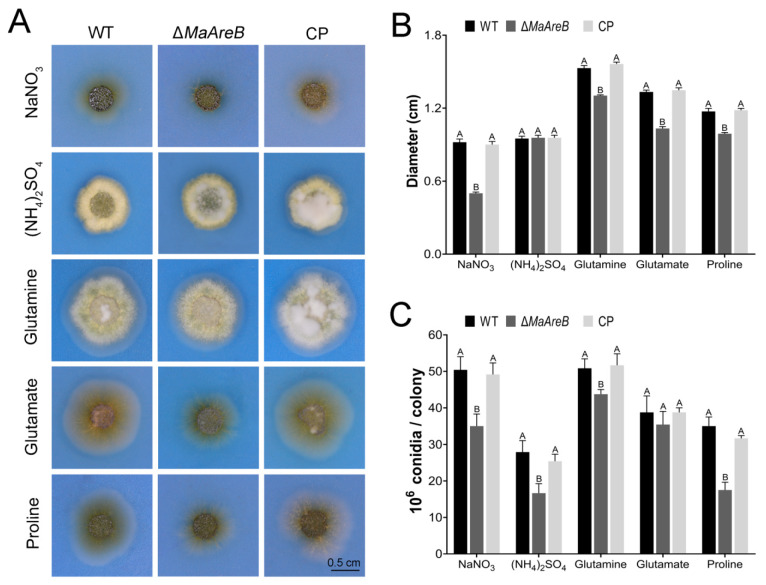
*MaAreB* was involved in regulating nitrogen utilization. Colony morphology (**A**), colony diameter (**B**) and conidial yield (**C**) of the WT, ∆*MaAreB* and CP strains on modified CZA and cultured at 28 °C for 7 days. The concentrations of all nitrogen sources were 10 mM. A&B, *p* < 0.05 (Tukey’s HSD). The *p*-values in Figure 2B,C were listed in supplementary Table S3.

AreB is a core member of NCR pathway and plays an important role in nitrogen utilization [14]. Thus, the WT, Δ*MaAreB* and CP strains were grown on modified CZA media, which were replaced the nitrate in CZA with 10 mM (NH_4_)_2_SO_4_, glutamine, glutamate or proline, respectively. The results showed that the colonies of ∆*MaAreB* grown on CZA media containing various nitrogen resources, except for ammonium, were smaller compared to the WT or CP strain (Figure 2A,B). The conidial yield of the ∆*MaAreB* strain decreased slightly with no significant difference to the WT or CP strain grown on glutamate as the sole nitrogen source, but was significantly decreased when nitrate, glutamine or proline was used as the sole nitrogen source (Figure 2C). These data evidenced that *MaAreB* was involved in regulating nitrogen utilization.

### 3.3. Deletion of MaAreB Affected Conidial Germination, Hyphal Growth and Conidiation

**Figure 3:**
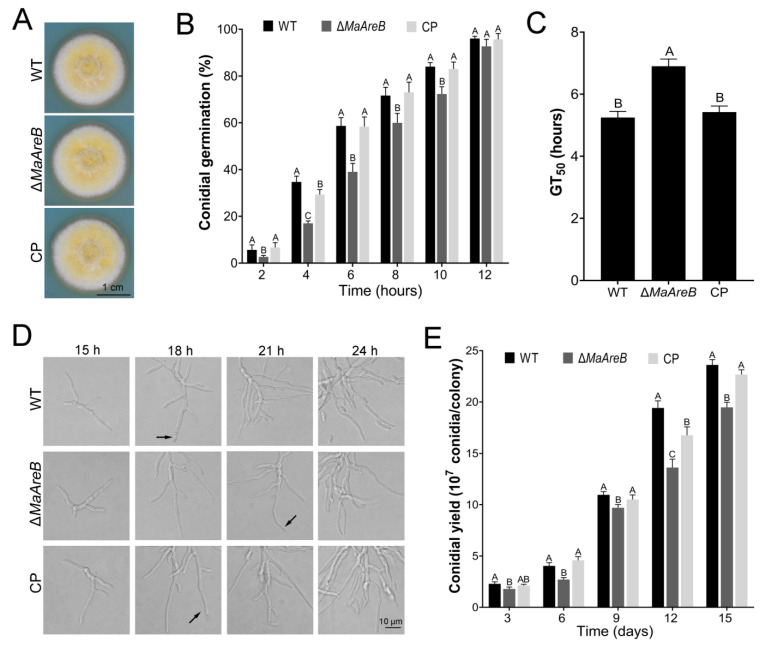
Deletion of *MaAreB* affected conidial germination, hyphal growth and conidiation. (**A**) Colonies of the WT, ∆*MaAreB* and CP strains cultured at 28 °C for 7 days. (**B**) The germination rates of each strain at different time points. The GT_50_ (**C**), microscopic observation of conidiation (**D**) and conidial yield (**E**) of each strain. All fungal strains were grown on 1/4SDAY. The arrows in Figure 3D indicated the conidium formed at the tip of the hypha. A&B, A&C and B&C, *p* < 0.05 (Tukey’s HSD). The *p*-values in Figure 3B,C,E are listed in supplementary Table S3.

To analyze the growth characteristics of the WT, ∆*MaAreB* and CP strains, we tested the conidial germinations, the macroscopic and microscopic growth morphologies, and the conidial yield of fungal strains grown on 1/4SDAY. The results showed that although there was no significant difference in colony morphology among the WT, ∆*MaAreB* and CP strains (Figure 3A), the germination rate of ∆*MaAreB* strain was significantly slower at 2, 4, 6, 8 and 10 h (Figure 3B). The GT_50_ (the mean 50% germination time) of the ∆*MaAreB* strain (6.90 ± 0.23 h) was significantly prolonged compared with the WT (5.25 ± 0.20 h) or CP (5.43 ± 0.19 h) strain (Figure 3C). Microscopic observation showed that the WT and CP strains began to yield conidia at 18 h, while the Δ*MaAreB* strain did at 21 h (Figure 3D). Furthermore, the conidial yield of the ∆*MaAreB* strain significantly reduced (Figure 3E). Taken together, it revealed that the *MaAreB* contributed to the conidial germination and the conidiation of *M. acridum*.

### 3.4. Deletion of MaAreB Weakened the Stress Tolerances to UV-B Irradiation and Heat-Shock

**Figure 4:**
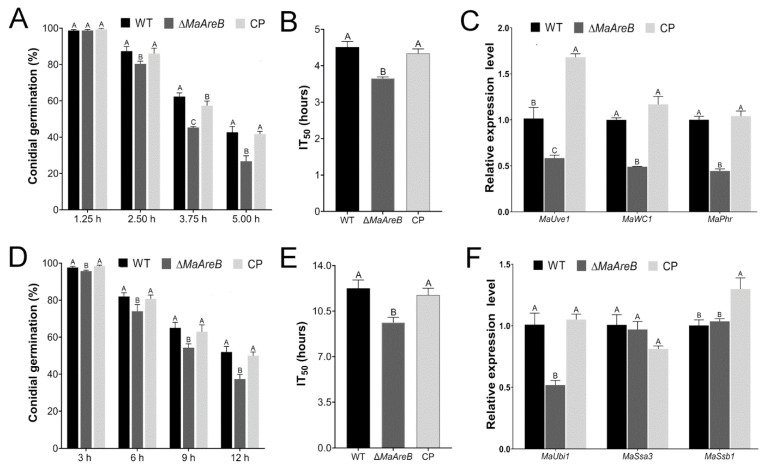
Deletion of *MaAreB* weakened tolerances to UV-B irradiation and heat-shock. Conidial germination of the WT, ∆*MaAreB* and CP strains with UV-B irradiation (**A**) and heat-shock (**D**) treatments. The IT_50_ of each strain with UV-B irradiation (**B**) and heat-shock (**E**) treatments. Transcription level of genes involved in fungal tolerances to UV-B irradiation (**C**) and heat-shock (**F**). A&B, A&C or B&C, *p* < 0.05 (Tukey’s HSD). The *p*-values in Figure 4A–F are listed in supplementary Table S3.

To clarify the effect of *MaAreB* on stress tolerances, the conidial germination rates of fungal strains were determined after treating with UV-B and heat-shock. These results showed that the conidial germination rate of ∆*MaAreB* was sharply decreased under UV-B treatment at 2.50 h, 3.75 h and 5.00 h (Figure 4A). The IT_50_ (mean 50% inhibition time) of the ∆*MaAreB* strain (3.65 ± 0.04 h) was markedly decreased compared with the WT (4.51 ± 0.16 h) or CP (4.34 ± 0.12 h) strain (Figure 4B). Furthermore, the transcription of the UV-B tolerance-related genes, *MaUve1*, *MaWC1* and *MaPhr*, were significantly decreased in Δ*MaAreB* (Figure 4C). After being treated with heat-shock, the germination rate of the ∆*MaAreB* strain also decreased significantly (Figure 4D). The IT_50_ of ∆*MaAreB* (9.62 ± 0.41 h) was significantly lower than that of the WT (12.26 ± 0.63 h) or CP (11.73 ± 0.53 h) strain (Figure 4E). In addition, the *MaUbi1* gene related to the heat-shock protection of fungus was significantly down-regulated in ∆*MaAreB* (Figure 4F). In summary, *MaAreB* played critical roles in fungal tolerances to UV-B irradiation and heat-shock.

### 3.5. Disruption of MaAreB Affected the Cell Wall Integrity

**Figure 5:**
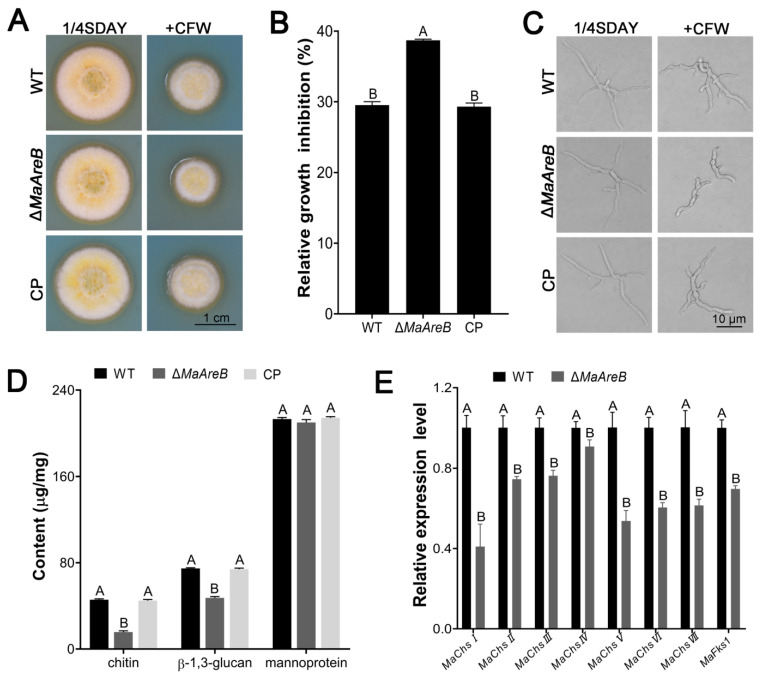
Disruption of *MaAreB* affected the cell wall integrity. (**A**) Growth of the WT, Δ*MaAreB* and CP strains on 1/4SDAY medium without or with 150 μg/mL CFW. (**B**) Relative growth inhibition rates. (**C**) Microscopic observation of hyphal growth for each strain on 1/4SDAY medium without or with CFW. (**D**) Determination of cell wall compositions for each strain. (**E**) Transcription levels of genes involved in the cell wall integrity. A&B, *p* < 0.05 (Tukey’s HSD). The *p*-values in Figure 5B,D,E are listed in supplementary Table S3.

The fungal cell wall plays crucial roles in fungal resistance to various adversities [42]. To further analyze the influence of *MaAreB* on the cell wall integrity of *M. acridum*, we added CFW into the 1/4SDAY to observe the growth of each strain. It was found that the colony of the Δ*MaAreB* strain was smaller than those of the WT and CP strains (Figure 5A), and the relative inhibition rate of the Δ*MaAreB* strain increased dramatically compared to the WT or CP strain (Figure 5B). Furthermore, the hyphal growth of the Δ*MaAreB* strain was significantly inhibited by CFW (Figure 5C*MaAreB* affected the cell wall integrity. Thus, the cell wall components of each strain were measured. As a result, the contents of chitin and β-1,3-glucan decreased significantly when the *MaAreB* gene was deleted, but the content of mannoproteins was not affected (Figure 5D). Meanwhile, some genes related to cell wall integrity, such as chitin synthase genes *MaChsI*, *MaChsII*, *MaChsIII*, *MaChsIV*, *MaChsV*, *MaChsVI*, *MaChsVII* and β-1,3-glucan synthase gene *MaFks1*, were significantly down-regulated in the Δ*MaAreB* strain (Figure 5E). Apparently, these results revealed that disruption of *MaAreB* affected the cell wall composition and structure of *M. acridum*.

### 3.6. Disruption of *MaAreB* Decreased Virulence

**Figure 6:**
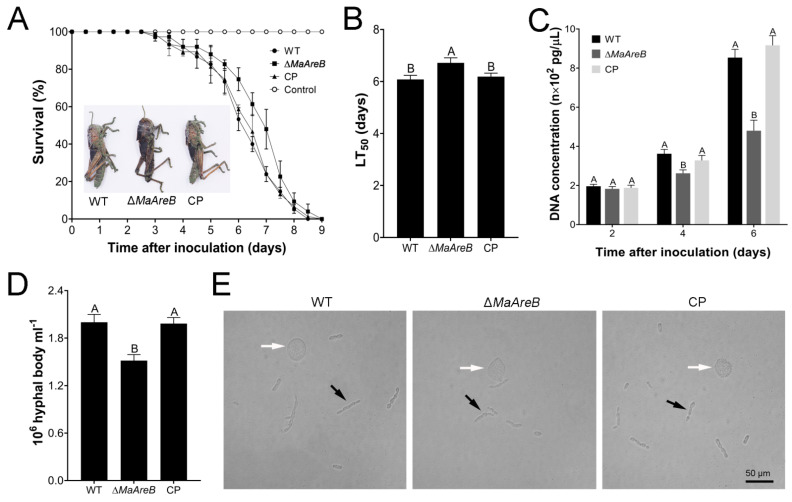
Loss of *MaAreB* reduced the virulence in topical inoculation. (**A**) Survival rates of locusts after infection with the WT, ∆*MaAreB* and CP strains in topical inoculation test, respectively. Locust cadavers killed by each strain were cultured at 28 °C for 8 days. (**B**) LT_50_s of fungal strains in topical inoculation test. (**C**) DNA concentrations of *M. acridum* in hemolymph of locusts infected with the WT, ∆*MaAreB* and CP strains for 2, 4 and 6 days in topical inoculation test. For each treatment, 600 μL blood was taken from 20 locusts (30 μL per locust) to extract the genomic DNA. The fungal DNA concentrations were determined using quantitative PCR with primer pair of ITS-F/ITS-R (Table S1) as described previously [39]. (**D**) The number of hyphal bodies in locust hemolymph by topical inoculation at 6 dpi. dpi, days post inoculation. (**E**) Microscopic images of hyphal bodies in locust hemolymph by topical inoculation at 6 dpi. The white arrows indicate the locust blood cells, and the black arrows indicate the hyphal bodies. A&B, *p* < 0.05 (Tukey’s HSD). The *p*-values in Figure 6B–D are listed in supplementary Table S3.

Bioassays were performed to test the virulence of the fungal strains through topical inoculation and intra-hemocoel injection. The topical inoculation experiment showed that the survival rates of locusts infected by the Δ*MaAreB* strain were higher than those of the WT or CP strain at the same infection time, and visualization of locust cadavers killed by the Δ*MaAreB* strain indicated less conidiation at 8 dpi (Figure 6A). The LT_50_ of the Δ*MaAreB* strain was 6.72 ± 0.11 d, which was significantly higher than that of the WT (6.08 ± 0.09 d) or CP (6.18 ± 0.08 d) strain (Figure 6B). Furthermore, the quantification of the DNA concentrations of hyphal bodies in the locust hemolymph was performed by quantitative PCR. As a result, the fungal DNA concentrations in locusts infected by the Δ*MaAreB* strain were markedly decreased at 4 dpi and 6 dpi compared to those infected by the WT or CP strain via topical inoculation (Figure 6C). Similarly, the number of hyphal bodies were also reduced greatly in those infected by the Δ*MaAreB* strain (Figure 6D,E). 

For the injection experiment, however, there was no significantly difference in the survival rates of locusts (Figure S2A), the LT_50_s (Figure S2B), the fungal DNA concentrations (Figure S2C) and the number of hyphal bodies in locusts among all fungal strains (Figure S2D,E). Taken together, these results indicated that loss of *MaAreB* severely affected the insect cuticle penetration process, reducing the virulence of *M. acridum*.

**Figure 7:**
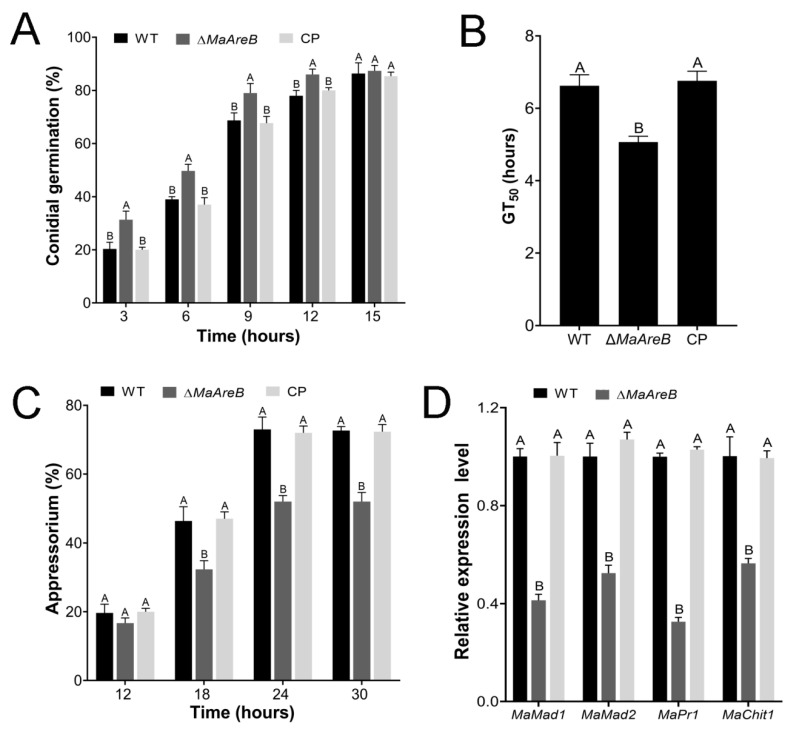
Lack of *MaAreB* impaired appressorium formation. The conidial germinations (**A**), GT_50_s (**B**) and appressorium formations (**C**) of the WT, Δ*MaAreB* and CP strains on the locust hind wings cultured at 28 °C for hours. (**D**) qRT-PCR analysis of genes involved in adhesion (*MaMad1* and *MaMad2*) and cuticle penetration (*MaPr1* and *MaChit1*). A&B, *p* < 0.05 (Tukey’s HSD). The *p*-values in Figure 7A–D are listed in supplementary Table S3.

Furthermore, the conidial germinations and appressorium formations of fungal strains were determined on the locust hind wings. The conidial germination was accelerated with the deletion of *MaAreB* (Figure 7A). The GT_50_ of the Δ*MaAreB* strain (5.07 ± 0.09 h) was significantly lower than that of the WT (6.62 ± 0.18 h) or CP (6.76 ± 0.15 h) strain (Figure 7B). However, the appressorium formation of the Δ*MaAreB* strain (52.00%) was heavily decreased compared to that of the WT (72.67%) or CP (72.33%) strain after 24 h (Figure 7C). qRT-PCR analyses showed that genes involved in fungal adhesion (*MaMad1* and *MaMad2*) and cuticle penetration (*MaPr1* and *MaChit1*) were significantly down-regulated in the Δ*MaAreB* strain (Figure 7D). The data indicated that loss of *MaAreB* affected the conidial germination and appressorium formation on the locust hind wings and the expression of genes related to adhesion and cuticle penetration.

**Figure 8:**
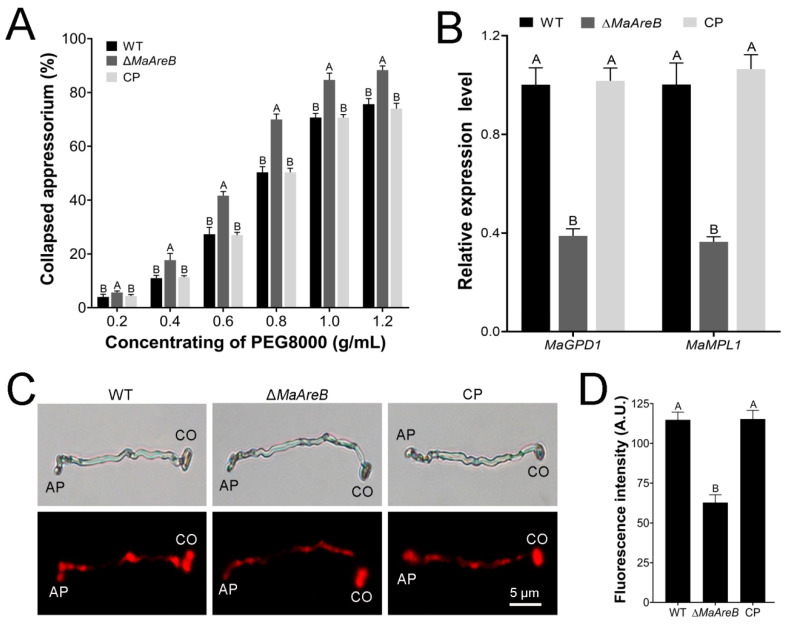
Lack of *MaAreB* decreased appressorial turgor pressure. (**A**) The appressorium collapsed rates of the WT, Δ*MaAreB* and CP strains after treatment with different concentrations of PEG8000. (**B**) qRT-PCR analysis of genes related to glycerol synthesis. (**C**) The neutral lipids stained using Nile red in appressoria. CO, conidium. AP, appressorium. Fungal samples were cultured on locust hind wings at 28 °C for 27 h. (**D**) Fluorescence intensity of the neutral lipids in appressoria. A.U., Arbitrary Units. A&B, *p* < 0.05 (Tukey’s HSD). The *p*-values in Figure 8A,B,D are listed in supplementary Table S3.

Turgor pressure is closely involved in the penetration of appressorium [9]. The appressorium collapsed rates of fungal strains were examined in different concentrations of PEG8000. The results showed that the appressorium collapsed rates of the Δ*MaAreB* strain were increased significantly compared with that from the WT or CP strain (Figure 8A). The glycerol produced by lipids in appressorium can enhance turgor pressure and promote the penetration of appressorium [41,43,44]. qRT-PCR analyses showed that the transcription of glycerol synthesis-related genes, *MaGPD1* and *MaMPL1* decreased significantly in Δ*MaAreB* (Figure 8B). Nile red staining demonstrated that the fluorescence intensity of the neutral lipids in appressoria of the Δ*MaAreB* strain was weaker than that in the WT or CP strain (Figure 8C,D).

## 4. Discussion

GATA transcription factors play vital roles in the NCR pathway, and regulate cell differentiation in different tissues [45]. The classical GATA transcription factor functions as a transcription activator and contains a transcription activation region at the N-terminal [45]. In filamentous fungi, only two GATA transcription factors, AreA and AreB, are found in the NCR pathway, among which AreA plays a positive regulatory role and AreB plays a repressive role [13,46]. AreA is a global transcriptional regulator related to nitrogen metabolism in filamentous fungi, which can activate the transcription of genes related to the secondary nitrogen metabolism, and it has been reported in many fungi, such as *A. nidulans* [47,48,49], *A. parasiticus* [50], *Gibberella fujikuroi* [51], *F. verticillioides* [26], and *F. fujikuroi* [18]. On the contrary, there are a few studies on *AreB* in filamentous fungi, which mainly focus on nitrogen metabolism and the regulatory relationship with NCR related genes [11,14,24]. In this study, we identified the AreB homologous protein, MaAreB, in the model insect pathogenic fungus *M. acridum*. The functions of MaAreB in nitrogen utilization, growth and development, tolerance to both, and virulence were characterized.

Based on the functions of GATA transcription factors in nitrogen metabolism, we explored the selective utilization of nitrogen sources by *MaAreB*. The results showed that the utilization ability of both preferential utilization glutamine and less easily assimilated nitrate were decreased in the Δ*MaAreB* strain. Although there was no significant difference in colony diameter on the ammonium, the conidial yield was significantly reduced in Δ*MaAreB*. These results suggested that *MaAreB* was also involved in regulating nitrogen utilization in *M. acridum*. Furthermore, loss of *MaAreB* resulted in a delay in conidial germination and a decrease in conidial yield when fungal strains were grown on the complete medium (1/4SDAY), indicating that *MaAreB* was involved in regulating the development and asexual sporulation.

*Metarhizium* spp. is a kind of important biocontrol fungus, which can infect a variety of agricultural pests [52,53,54,55,56]. The adaptability of entomopathogenic fungi to the adverse environment directly determine the efficacy of mycoinsecticides [57]. In this study, we found that loss of *MaAreB* reduced the tolerances to UV-B irradiation and heat-shock in *M. acridum*. As a powerful physiological barrier, the fungal cell wall involves a variety of physiological functions, such as nutrient uptake and cell attachment, maintaining the inherent morphology of fungal cells, and protecting cells from external adversity conditions [58,59,60,61]. Our results showed that *MaAreB* affected the cell wall integrity of *M. acridum*. The hyphal growth and colony morphology of the Δ*MaAreB* strain were seriously inhibited by CFW, which could bind to the nascent chitin chain to prevent the formation of co-crystals and microfibrils [62]. Chitin is synthesized by chitin synthases and is crucial to the fungal cell wall integrity [63]. Studies have shown that *ChsIII*, *ChsV*, *ChsVI* and *ChsVII* affect the synthesis of polysaccharide in the cell wall, and *ChsIII*, *ChsV* and *ChsVII* are necessary for the cell wall integrity in *M. acridum* [36]. In addition, chitin syn synthases in *A. fumigatus* [64] and *Botrytis cinerea* [65] also affect the cell wall integrity. We found that the contents of chitin and β-1,3-glucan, the essential components of the fungal cell wall, in Δ*MaAreB* strain were heavily decreased. Consistently, some genes encoding chitin synthases or β-1,3-glucan synthase were significantly down-regulated. Therefore, we speculated that *MaAreB* might make contributions to the fungal sensitivity and resistance to the external environment by affecting the cell wall integrity.

Studies in many fungi, such as *Ustilago maydis* [66], *M. oryzae* [67], and *A. fumigatus* [64,68], have pointed out that the change of chitin content in the cell wall affects appressorium formation, which in turn affects the host immune defense. In this study, we found that disruption of *MaAreB* affected the synthesis of fungal cell wall components and cell wall integrity. The virulence test showed that the virulence of the Δ*MaAreB* strain was significantly decreased in topical inoculation, while it was not significantly different from that of the WT or CP strain in intra-hemocoel injection, indicating that *MaAreB* was conducive for *M. acridum* to penetrate the host cuticle. The ability of conidium to penetrate the host cuticle was directly determined by its adhesion, germination, and appressorium formation on the host cuticle [8]. Our study proved that the appressorium formation, the appressorium turgor pressure, and the transcription of genes related to adhesion (*MaMad1* and *MaMad2*) and penetration (*MaPr1* and *MaChit1*) were all significantly reduced in the Δ*MaAreB* strain. Appressoria of entomopathogenic fungi are necessary for infecting host, and the turgor pressure and cuticle degrading enzymes can promote appressoria to penetrate the host cuticle [9]. These data further confirmed that *MaAreB* played an important role in insect cuticle penetration of *M. acridum*.

Nitrogen metabolism is not only involved in regulating the nutrient utilization of fungi, but also closely involved in the virulence of pathogenic fungi. Glutamine, a preferential nitrogen source, could inhibit the virulence of *M. oryzae* [28,69] and *F. oxysporum* [70]. Deletion of *MoGln1*, the glutamine synthetase gene, would affect the virulence of infecting hyphae in *M. oryzae* [69]. During the process of host infection by entomopathogenic fungi, conidial germination [71,72] and appressorium formation [73] are closely related to nitrogen source conditions. *B. bassiana* needs to activate its own intracellular nitrogen metabolism level to promote conidial germination; if not, the conidia cannot form germ tubes [74]. Nitrogen starvation can promote appressorium formation in *M. anisopliae* [75]. Nitrogen starvation can induce the expression of many pathogenesis related genes; hence it is also regarded as the driving force for pathogenic fungi to infect the host [76,77]. In *M. oryzae*, deletion of *Asd4*, the *AreB* homologous gene, could increase glutamine content and activate TOR (Target of Rapamycin) pathway, and result in the inability to form appressorium [28]. However, the appressorium formation could be restored with knocking out *Gln1* or inhibiting TOR path under Δ*Asd4* background [28]. In *M. acridum*, *MaAreB* was involved in regulating nitrogen metabolism and virulence, but it was still unclear whether there was a certain correlation between nutrient utilization and virulence. Whether *MaAreB* affects virulence by inducing nitrogen metabolism remains to be explored in the near future.

## 5. Conclusions

Nutrients are very important for fungal growth and development. It is well known that the NCR pathway plays crucial roles in the regulation of fungal nitrogen metabolism. During these processes, GATA-type transcription factors, such as AreA and AreB, play indispensable roles. Although AreA has been reported in many fungi, the second GATA transcription factor AreB is less studied than AreA. The functions of AreB are largely unknown in entomopathogenic fungi. In this study, we focused on functional characterization of the second GATA transcription factor MaAreB in the model entomopathogenic fungus *M. acridum*. Deletion of *MaAreB* affected the fungal cell wall integrity and reduced fungal virulence due to weakening the cuticle penetration of *M. acridum*. However, the roles of *MaAreB* in nitrogen utilization needs to be further investigated in *M. acridum*. In general, these results provide insights into understanding the functions of AreB homologous proteins in other filamentous fungi.

## Data Availability

Not applicable.

## Supplementary Materials

The following are available online at https://www.mdpi.com/article/10.3390/jof7070512/s1, Figure S1: Vector construction and Southern blotting. Schematic diagram of *MaAreB* knockout (A) and complemented (B) vector constructions. (C) Verification of the WT, Δ*MaAreB* and CP strains by Southern blotting. Probe was amplified with primers AreB-PF/AreB-PR (Table S1). Restriction enzyme *Eco*RV and *Sac*II were used to digest the genome DNA of the WT, Δ*MaAreB* and CP strains. Figure S2: Loss of *MaAreB* did not affected the virulence in intra-hemocoel injection. (A) Survival rates of locusts infected with the WT, ∆*MaAreB* and CP strains in intra-hemocoel injection, respectively. Locust cadavers killed by fungal strains were cultured at 28 °C for 8 days. (B) LT_50_s of fungal strains in intra-hemocoel injection test. (C) DNA concentrations of *M. acridum* in hemolymph of locust infected with the WT, ∆*MaAreB* and CP strains for 1 d, 3 d and 5 d in intra-hemocoel injection test. For each treatment, 600 μL blood was taken from 20 locusts (30 μL for a locust) to extract the genomic DNA. The fungal DNA concentrations were determined using quantitative PCR with primer pair of ITS-F/ITS-R (Table S1) as described previously [39]. (D) The number of hyphal bodies in locust hemolymph by intra-hemocoel injection at 5 dpi. (E) Microscopic images of hyphal bodies in locust hemolymph by intra-hemocoel injection at 5 dpi. dpi, days post inoculation. The white arrows indicate the locust blood cells, and the black arrows indicate the hyphal bodies. A&A, *p* > 0.05 (Tukey’s HSD). The *p* values in Figure S2B–D were listed in supplementary Table S3. Table S1: Primers used in this study. Table S2: Parameter information of primer pairs used in qRT-PCR. Table S3: The *p*-values in each experiment.
