# Supplementary material for: MaAreB, a GATA Transcription Factor, Is Involved in Nitrogen Source Utilization, Stress Tolerances and Virulence in Metarhizium acridum
Source: J Fungi (Basel). 2021 Jun 27;7(7):512. doi: 10.3390/jof7070512 (PMC8305397; doi:10.3390/jof7070512)
Supplement: Supplementary file 1 [file jof-07-00512-s001.zip › Supporting Figures.pdf]

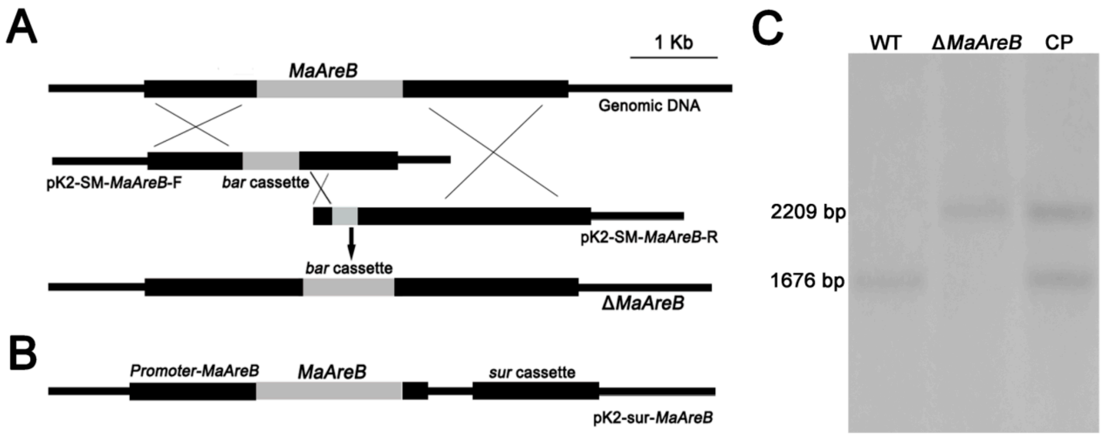

Figure S1 Vector construction and Southern blotting verification. Schematic diagram of *MaAreB* knockout (**A**) and complemented (**B**) vector constructions. (**C**) Verification of the WT, Δ*MaAreB* and CP strains by Southern blotting. Probe was amplified with primers AreB-PF/AreB-PR (Table S1). Restriction enzyme *Eco*RV and *Sac*II were used to digest the genome DNA of the WT, Δ*MaAreB* and CP strains.

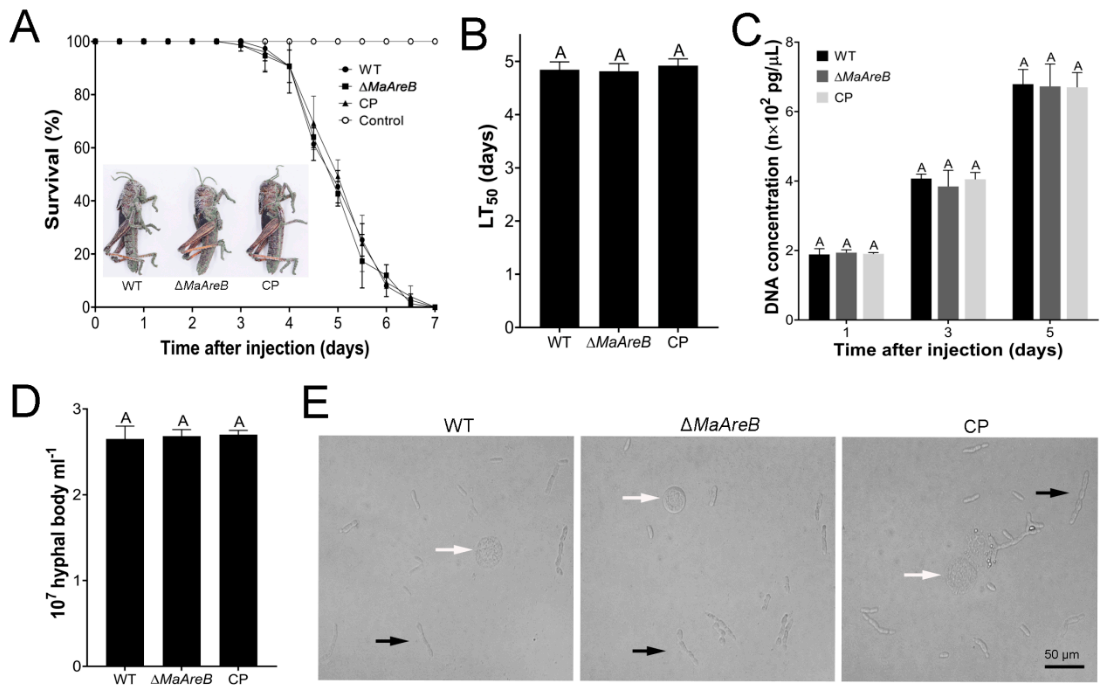

Figure S2 Loss of *MaAreB* did not affected the virulence in intrahemocoel injection. (A) Survival rates of locusts infected with the WT, Δ*MaAreB* and CP strains in intrahemocoel injection, respectively. Locust cadavers killed by fungal strains were cultured at 28℃ for 8 days. (B) LT_{50}s of fungal strains in intrahemocoel injection test. (C) DNA concentrations of *M. acridum* in hemolymph of locust infected with the WT, Δ*MaAreB* and CP strains for 1 d, 3 d and 5 d in intrahemocoel injection test. For each treatment, 600 μL blood was taken from 20 locusts (30 μL for a locust) to extract the genomic DNA. The fungal DNA concentrations were determined using quantitative PCR with primer pair of ITS-F/ITS-R (Table S1) as described previously [39]. (D) The number of hyphal bodies in locust hemolymph by intrahemocoel injection at 5 dpi. dpi, days post inoculation. (E) Microscopic images of hyphal bodies in locust hemolymph by intrahemocoel injection at 5 dpi. The white arrows indicate the locust blood cells, and the black arrows indicate the hyphal bodies. A&A, $P > 0.05$ (Tukey's HSD). The *p*-values in Figure S2B-D were listed in supplementary Table S3.
